# The Improvement of Trunk Muscle Endurance in Adolescents with Idiopathic Scoliosis Treated with ScoliBrace^®^ and the ScoliBalance^®^ Exercise Approach

**DOI:** 10.3390/jcm13030653

**Published:** 2024-01-23

**Authors:** Rosemary Marchese, Juan Du Plessis, Tamara Pooke, Jeb McAviney

**Affiliations:** ScoliCare, Sydney, NSW 2217, Australia; juan@scolicare.com (J.D.P.); tammy.meuwese@scolicare.com (T.P.); jeb@scolicare.com (J.M.)

**Keywords:** trunk muscle, endurance, adolescents, idiopathic scoliosis, exercise, scoliosis

## Abstract

The impact of scoliosis bracing combined with physiotherapeutic scoliosis-specific exercises (PSSE) on trunk muscle endurance in adolescents with idiopathic scoliosis is unknown. ScoliBrace^®^, a rigid, three-dimensional, over-corrective thoraco-lumbar-sacral orthosis (TLSO), and ScoliBalance^®^, a PSSE program, were used to treat adolescent idiopathic scoliosis (AIS) patients. A retrospective study of the trunk muscle endurance of 33 AIS patients who received ScoliBrace^®^ and ScoliBalance^®^ was conducted. The patients were treated with ScoliBrace^®^ and an individualized ScoliBalance^®^ program. Trunk extensor muscle endurance (TE) and abdominal muscle endurance (AE) tests were performed at initial assessment and then at averages of 6.6 and 24.4 weeks of treatment. The data were analyzed using the Wilcoxon signed-rank test, Stata version 15.1. The participants were aged 13.24 years (SD = 1.64) with a mean Cobb angle of 38.97° (SD = 9.49°). TE improved significantly (*p* < 0.001) at both short- and medium-term intervals using ScoliBalance^®^ and ScoliBrace^®^ in the AIS patients. AE also showed significant improvement between baseline and short-term follow-up, with non-significant improvement at medium-term follow-up. Overall, trunk muscle endurance showed improvement in the AIS patients using ScoliBrace^®^ and ScoliBalance^®^. Future research is required to determine the individual and combined effects of each treatment. However, it seems likely that trunk muscle endurance will not deteriorate in AIS patients with this combined treatment.

## 1. Introduction

Adolescent idiopathic scoliosis (AIS) is a condition that results in a lateral deviation of the spine combined with vertebral rotation, with a Cobb angle of ≥10° Cobb on X-ray, occurring in otherwise healthy individuals and often diagnosed at or around the time of puberty [1]. The condition is more common in females, with an overall prevalence of 0.47% [2]. The risk of progression is linked to the initial curve magnitude and the remaining growth [3]. Most of the potential negative consequences of AIS manifest once the curve exceeds 30 degrees [4]. Curves that remain under 30 degrees at skeletal maturity are unlikely to progress during adulthood [5].

Treatments for AIS include scheduled observation/monitoring, physiotherapeutic scoliosis-specific exercises (PSSE), bracing, and surgery [6]. PSSE is recommended as either a standalone treatment in mild AIS, as an adjunct to bracing in patients with moderate to severe AIS, and during the brace weaning process [7,8,9,10]. The combination of bracing combined with PSSE has been shown to have better outcomes in AIS with curves of 25–40 degrees [8].

PSSE should consist of the following: auto-correction in 3D, stabilizing the corrected posture, training in activities of daily living (ADL), and patient education [7]. There are various schools worldwide that offer training in various types of PSSE, each founded differently, but all claiming to incorporate the recommended four components of PSSE [11].

The evidence in support of PSSE for AIS has been evolving, but the efficacy of exercise treatment remains controversial [12,13,14,15,16]. A survey of Scoliosis Research Society members showed that only 22% of respondents use PSSE for AIS, with skepticism remaining regarding the benefits of PSSE for AIS [17].

The publication of the BRAiST study, which demonstrated the efficacy of bracing for AIS based on clearly defined clinical parameters, has been pivotal in the changing of attitudes towards the support of bracing for AIS [18]. However, some skepticism remains towards bracing for AIS in regard to any potential side effects such as restriction in movement, with the rigidity of bracing thought to possibly reduce core muscle usage and strength and ultimately peak bone mass development [19]. However, so far, this fear seems unfounded, with a recent review and meta-analysis on the impact of the continuous use of a lumbosacral orthosis for musculoskeletal conditions for a period of 1–6 months showing no negative effects [20].

The potential influence of scoliosis treatments on both the anterior and posterior trunk musculature in AIS is an important clinical consideration, particularly considering that reduced trunk muscle endurance is thought to contribute to the development of low back pain in the general population [21]. Furthermore, abdominal trunk muscle weakness has also been reported in older women with chronic low back pain and is associated with a history and risk of falling; it is therefore logical that any trunk muscle weakness is addressed in AIS. A recent analysis of the trunk muscle strength in a group of AIS patients demonstrated weaker trunk extensors and flexors compared to healthy females. The authors concluded that, within the analyzed AIS population, weight and body mass indexes appear to have a negative impact on muscular performances, whereas the clinical and radiological characteristics of the scoliosis do not seem to contribute [22]. AIS patients have been reported to display asymmetrical changes in their paravertebral muscles, namely, changes in myoelectric activity, perfusion, and increases in Type I muscle fibers on the side of the convexity [23,24,25]. The spinal musculature is reported as being the most affected on the concave side of the curve near the apex, with the muscles adopting a ‘faster’ or more ‘glycolytic’ profile, indicating reduced, low-level tonic activity of the muscle [26]. A difference in abdominal muscle thickness, as measured by ultrasound, has also been seen between healthy adolescents and those with idiopathic scoliosis [27,28,29,30]. In a comparison study between women with scoliosis and healthy controls, those with scoliosis with a Cobb angle of greater than 30 degrees showed more trunk weakness than the healthy counterparts [31].

There have been varying reports regarding the potential impacts of bracing on the trunk muscles in AIS patients and the non-AIS population. An electromyographical (EMG) study conducted in this area indicated that, immediately following the application of a brace (Boston type), there were significant increases in back muscle EMG activity in less than half (43%) of patients and the brace did not seem to have any effect on the abdominal muscles [32]. AIS patients treated with either surgery (Harrington technique) or bracing (Milwaukee and Boston types) have been reported to have reduced long-term (20 years) trunk muscle endurance [33]. A systematic review evaluating whether lumbosacral orthoses (not individually customized) resulted in trunk muscle weakness or atrophy in healthy subjects or patients with low back pain did not find any conclusive evidence to suggest that orthoses resulted in trunk muscle weakness [34]. A randomized controlled trial (RCT) showed that the use of a soft orthosis on healthy individuals increased muscle strength after 21 days of use [35].

Until recently, the influence of various available non-surgical scoliosis treatments on the unique trunk musculature in this AIS patient population has been largely unknown However, there is now evidence that the addition of PSSE to bracing may result in increased trunk extensor muscle endurance (TE) compared to orthotic intervention (TLSO or Boston type) alone [8,13]. Integrating orthotic intervention with scoliosis-specific exercises has shown better outcomes in terms of Cobb angle, respiratory parameters, and back muscle endurance than the use of an orthosis alone [8]. A long-term follow-up study showed that patients with early-onset scoliosis treated with a brace had a similar trunk muscle endurance to untreated patients with AIS and a significantly better trunk muscle endurance than adolescents with scoliosis treated with a brace [36]. The findings of this study show that those with earlier onset scoliosis and a longer bracing duration have better outcomes in range of motion and trunk muscle endurance [36]. There are currently no other publications for comparison. The aim of our study was to retrospectively review the short- and medium-term TE and abdominal muscle endurance (AE) measurements of AIS patients undergoing brace treatment using ScoliBrace^®^ as a rigid 3D brace in combination with a ScoliBalance^®^ exercise program as the chosen PSSE.

## 2. Materials and Methods

### 2.1. Sample

This retrospective study was performed using a database of 98 AIS patients who were treated using ScoliBalance and ScoliBrace at a single site in Sydney, Australia, between 2014 and 2016 and met the study inclusion criteria. Of this sample, 33 patients (33.67%) consented to having their data utilized for research purposes.

### 2.2. Inclusion and Exclusion Criteria

The patients included fulfilled the following criteria: participated in ScoliBalance^®^ as a PSSE program (Appendix A), where the principles of PSSE are incorporated into each patient program; underwent their baseline assessment prior to or on the day of the brace fitting; treated full-time using the rigid TLSO called ScoliBrace^®^ (ScoliCare Pty Ltd. Sydney, Australia) (Appendix B); and underwent their initial follow-up assessment ≥4 weeks after their brace fitting [7]. The inclusion criteria: AIS patients receiving ScoliBrace^®^ and ScoliBalance^®^ treatment. The exclusion criteria: spinal surgery and scoliosis of >60°.

The principles behind the design, manufacture, and action of this orthosis have been described elsewhere [37,38]. Briefly, ScoliBrace^®^ is a customized, rigid, over-corrective TLSO designed to place the patient in an in-brace position that attempts to correct the spine and posture in three dimensions using a “Mirror Image^®^” (with permission) approach [37,38,39]. Each ScoliBrace^®^ is custom designed and made for the patient with the latest in 3D scanning technology and computer-aided design and manufacture (CAD CAM) (Canfit, V17), using a specific design algorithm and patient-centered approach to bracing treatment. The ScoliBrace^®^ uses an over-corrective approach to guide the body into a posture that is the opposite to the way the scoliosis has positioned it, with the aim of reducing the Cobb angle where possible. By putting the body posture in this over-corrected position, it forces the spine to begin to straighten using the concept of spinal coupling, i.e., as the body moves into the opposite position, the spine moves with it towards that position, achieving the maximum straightening within the limits of the spine’s flexibility. The patients were prescribed to wear this orthosis full-time (20–23 h per day).

Participants were also prescribed a ScoliBalance^®^ program, delivered by ScoliBalance^®^-trained therapists who were working in a multi-disciplinary team. ScoliBalance is a physiotherapeutic scoliosis-specific exercise rehabilitation program (PSSE) used to treat scoliosis and kyphosis in young children, adolescents, and adults. The aim for each patient is to achieve 3D auto-correction of their posture as early in the program as possible. Each patient is given a progressive, individualized program according to their curve type, clinical presentation, and treatment goals. The programme incorporates the principles of PSSE and combines the most relevant practices and principles of physical therapy, chiropractic, and exercise rehabilitation [7]. ScoliBalance^®^ builds on the foundation of other PSSE programs, incorporating a focus on achieving a specific postural correction (ScoliCorrection) for a patient’s curve type and posture, and then strengthening that correction using a specific set of exercises (ScoliExercises). The ScoliCorrections are initially taught in a seated position, incorporating corrections of the pelvis and trunk positions and harnessing the principles of spinal coupling and detorsion. The ScoliExercises may incorporate a variety of specifically chosen exercises adopted or adapted from various PSSE schools. Examples include sets of functional challenges for the patient to learn to hold their corrected posture for sustained periods of time. The patient may be taught corrective breathing or rotational angular breathing to enhance the axial expansion in more collapsed areas of the trunk if there is still trunk collapse after the ScoliCorrection is attempted by the patient [11]. A specific set of stabilization exercises, e.g., the use of isometric resistance using poles, may also be incorporated as a strengthening strategy. The patient is taught to integrate their ScoliCorrections into activities of daily living (ADLs) from the very first rehabilitation session, learning to also incorporate visualization and other strategies to enhance their adoption of the corrected posture. A daily home exercise program and ADL integration strategies are prescribed for all patients.

The AIS patients were taught their program during one-hour, one-on-one rehabilitation sessions with a ScoliBalance^®^ Provider who had undertaken specific ScoliBalance^®^ education, training, and assessment. The initial scheduling of sessions was typically frequent (e.g., weekly) as a patient was learning the ScoliCorrections and ScoliExercises. The patients were instructed to continue performing the various exercises and associated progressions at home for 15–20 min per day in addition to the ScoliBrace^®^ treatment. The exercises were performed out-of-brace. They were also taught to integrate their ScoliCorrection into their ADLs. Performance and compliance of the exercise programme were monitored at all the exercise sessions held in the clinic, where any issues were addressed.

### 2.3. Data Collection and Outcome Measures

The primary outcome of interest for the review was trunk muscle endurance, in particular TE and AE. TE and AE times were assessed prior to the brace fitting and then again at two subsequent short- and medium-term follow-up points. These were assessed using the clinically applied Beiring–Sorenson Test and the V-sit test [21,40].

**Figure 1:**
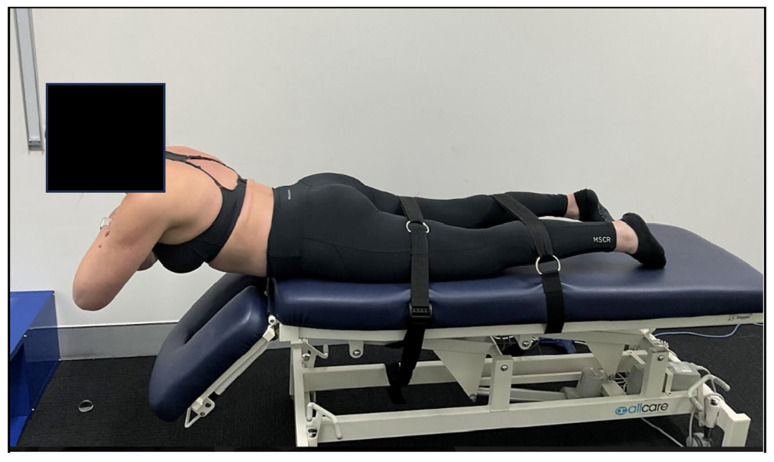
Biering–Sorensen Test for trunk extensor endurance used to assess trunk muscle extensor endurance in adolescents with idiopathic scoliosis (AIS) who were being treated with ScoliBalance^®^ and ScoliBrace^®.^

The Biering–Sorensen test was used to assess TE. The test involved recording how many seconds a patient could keep their unsupported upper body horizontal while in a prone position [21]. The Biering–Sorensen test assesses endurance via the isometric contraction of the trunk extensors in a cantilevered position on a bench or standard plinth. Figure 1, Biering–Sorensen Test.

In preparation for the test, the lower half of the body was anchored to a bench using two or three straps (the third strap just inferior to the anterior superior iliac spines). The straps were positioned and comfortably fastened for the patient. The patient was asked to rest their arms and the weight of their trunk on a chair placed in front of the bench during the setup procedure. To begin the test, the participant was instructed to lift their upper body up so that it was parallel with the ground, or just above, hold their arms folded across their chest, and maintain this position for as long as possible. The examiner then measured the time that the participant could maintain the test position using a stopwatch. The test was terminated in the event that either: the participant’s upper trunk dropped more than 5–10° below horizontal; the participant maintained the test position for the maximum time (180 s); or the provocation of pain or other symptoms by the test position prevented the participant from continuing the test. The patients were given verbal feedback from the examiner during the test if their upper body was dropping below parallel and given one opportunity to return to neutral position and continue the test.

**Figure 2:**
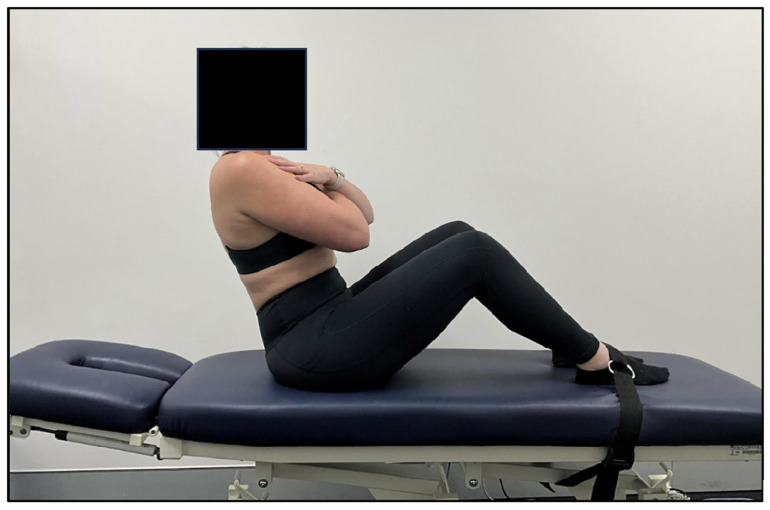
V-Sit Test for abdominal endurance used to assess trunk muscle extensor endurance in adolescents with idiopathic scoliosis (AIS) who were being treated with ScoliBalance^®^ and ScoliBrace^®.^

The V-sit test was used to assess AE. The test involved recording how many seconds a patient could keep their unsupported upper body reclined at an angle of 60° above the horizontal plane, while the knees and hips were kept flexed to 90° and the feet were anchored to a bench using a strap [40]. The patient sat on the bench in an upright posture prior to commencing the test. The patients were then instructed to cross their arms across their chest, maintain a neutral spine, and lean their body back until it was in line with a wall marker that was set at 60° above the horizontal plane (Figure 2). The examiner measured the time that the patient could maintain this test position using a stopwatch. The V-sit test was ceased if: the participant’s upper trunk dropped more than 5–10° below the wall marker; the patient maintained the test position for the maximum time (180 s); or the provocation of pain or other symptoms by the test position prevented the participant from continuing the test. The patients were provided with verbal feedback from the examiner during the test if the angle of their trunk was dropping below the 60° wall marker and given one opportunity to restore their body angle and continue the test.

Both the Biering–Sorensen test and V-sit test were assessed by one of two ScoliBalance^®^ Providers who have clinical experience in administering these tests to AIS patients. Longer hold times, up to a maximum of 180 s, are indicative of better results on both the tests. The patients were not provided with any clues to their times until the conclusion of each of the tests.

In addition to the TE and AE times, each patient’s: age; gender; curve type; and the initial Cobb angle of their primary curve were recorded. The authors also logged the date of the brace fitting, as well as the dates of each of the follow-up assessments.

### 2.4. Analysis

Descriptive statistics were produced for all clinical and demographic variables. Mean and standard deviation (SD) are reported for patient demographics, and median and interquartile range (IQR) are presented for clinical assessment, where data were not normally distributed. The data analysis was conducted in Stata version 15.1.

## 3. Results

### 3.1. Initial Description of the Dataset

A total of 33 AIS patients were originally recruited for this study. A small number of patients did not have their TE or AE recorded at the short-term or medium-term follow-up timepoints. Times were recorded up to a maximum of 180 s. The demographics of these 33 patients are shown in Table 1.

### 3.2. Comparing TE and AE between Timepoints

#### 3.2.1. TE and AE including All Cases (Including Those Patients Who Achieved the Maximum of 180 s)

This analysis included the patients who achieved the maximum of 180 s. Not all patients had measurements recorded at all follow-up timepoints, which is why only 28 of the initial 33 patients were included in the follow-up analysis. A Wilcoxon signed-rank test was used to determine if there was a significant difference in the timing of the tests between the timepoints, as the data were not normally distributed. An increase was seen in all median times, showing that there were improvements in TE and AE. However, only TE showed a significant improvement from baseline at short- and medium-term follow-ups, along with AE between baseline and short-term follow-up. The median of the difference between the two timepoints was calculated, along with a 95% confidence interval using a binomial method. Note that the median of the differences is not necessarily equal to the difference of the medians. The results for this are seen in Table 2.

#### 3.2.2. TE and AE (Not including Patients Who Achieved the Maximum of 180 s)

Table 3 shows the analysis of the data only for the patients who did not achieve the maximum of 180 s. Note that not all patients had measurements recorded at the follow-up timepoints for TE (*n* = 12) and AE (*n* = 11). A Wilcoxon signed-rank test was used to determine if there was a significant difference between the timepoints.

### 3.3. Number and Proportion of Those Who Reached 180 s

The number and proportion of patients who reached 180 s for TE and AE at the three timepoints can be seen in Table 4.

## 4. Discussion

This review showed significant short-term and medium-term improvements in trunk muscle endurance in AIS patients undergoing ScoliBalance^®^ and ScoliBrace^®^ treatment, especially when including those patients that could achieve the maximum timing for each of the tests. When excluding those patients that achieved the maximum of 180 s at all time-points, improvement was still seen, but was only significant for the difference between TE at baseline and short-term follow-up. This may have been due to the small remaining sample size after a large proportion of the sample were able to achieve the maximum possible result on the chosen trunk muscle endurance test. It is possible that further improvements may have been seen if the tests continued beyond the chosen maximum time point. Future studies may look to increase the maximum timepoint to more than a 180 s duration.

The baseline findings in our patients who did not reach the maximum showed a median TE of 70 s, consistent with the findings of other authors [41]. This earlier study examined back muscle endurance in a birth cohort of 1435 Australians at age 14 and found a mean hold time on the Beiring–Sorensen test of 83 s (SD = 62.7) [41]. More recently, norms for TE were reported as a mean of 181.0 s (±66.8) for males and a mean of 183.2 s (±92.8) for females, which are longer than those seen in the TE baseline times in our study [42]. However, these means were established from 20 volunteers aged 40.9 ± 11.6 years old, ranging in age from 21 to 58 years, who had no history of acute or chronic back disease, and therefore were not representative of our study sample [42].

The post-treatment medians in this study are mostly consistent with those reported in the literature [41,42,43,44,45,46]. For TE, a cross-sectional study of 9413 Danish school students (mean age of 17 years, SD = 0.6) was 147.9 s (SD = 57.3) for males and 148.9 s (SD = 66.9) for females [47]. Normative data constructed for TE for a sub-Saharan African population showed that the mean TE in individuals aged 11–19 years was 150 s for males and 127 s for females [45]. A study of 625 adolescents in Nigeria, with a mean age of 13.5 years (SD = 1.6), reported that the mean hold time for TE was 132.9 s (SD = 65.7) [44]. It can be seen from the literature that there is variation in the mean hold times for trunk endurance in various populations. It is also important to note that none of these established means were determined from AIS patients. The variation from our baseline means may possibly be explained by the alterations in the spinal biomechanics in patients with scoliosis [48,49]. The reduced TE times may because of pain or abnormal loading/force of the trunk muscles [50,51].

For baseline AE, our AIS patients started with a median of 95 s (Table 2). Excluding the patients that were able to reach the maximum threshold of 180 s reduced this median slightly to 81.5 s (Table 3). The literature shows varied means ranging from 101 to 123 s in adult populations without scoliosis [42,43,46]. Again, it is possible that the trunk changes in AIS patients were the reason for the lower baseline performance of our patients, and the influence of age cannot be determined from these data.

The findings in our study show significant improvement in AE between baseline and at short-term follow up, with a median maximum score of 180 s. This may explain why the times measured between short-term and medium-term, although improved, were non-significant. When removing patients that achieved the maximum of 180 s (*n* = 24), longer hold times were still seen between baseline and follow-ups, but they were not of statistical significance. This may have been due to the smaller number of patients analyzed at the short-term (*n* = 12) and medium-term (*n* = 11) follow-ups.

Without a control group, and with not much research on this topic, it is unclear as to what happens to trunk muscle endurance in AIS patients treated with bracing alone. This is important in that there have been concerns raised that bracing may result in deleterious alterations in trunk muscle function; however, there is no strong evidence to support such claims [9,33,52].

An RCT studied the effects of adding exercise to the standard of care in a group of 50 AIS patients with mild to moderate curves [13]. In this study, muscle endurance, as measured by the Biering–Sorenson test, showed improvement from baseline in both control (observation and bracing) and treatment (Schroth, observation and bracing) groups, with the addition of the exercises resulting in longer hold times at three months. However, there was no significant change in the hold times between groups from three to six months of treatment [13]. The results in their study are somewhat comparable to our findings, in that there were recorded increases in the trunk muscle endurance times in each of the two follow-up assessments, with only the increase from baseline to initial follow-up showing statistical significance. In an RCT, an orthotic orthosis and scoliosis-specific exercise showed better results at 6-month follow-up than the use of an orthosis alone in terms of spinal deformity, back muscle endurance, and pulmonary function [8].

### Limitations

Our retrospective study contributes to the scant literature on the short–medium-term trunk muscle endurance of AIS patients undergoing bracing and PSSE. Although standardized and reliable testing procedures were applied by a team of ScoliBalance^®^-trained therapists, this study was limited by not being able to control the timing of the follow-up assessments. A limitation of the study was that it was conducted as a retrospective analysis of consenting patients, so we were not able to control all parameters, including patient compliance and exercise performance. In future studies, a more prescribed assessment protocol can be established at set timeframes. Another limitation of this study is the small sample size. With the results of this retrospective study showing promise, a larger prospective study is justified. The authors also suggest a future larger prospective cohort study with a control group for comparison, as well as the inclusion of compliance monitoring and recording strategies to be able to accurately assess any influence of compliance and motivational strategies on the results. Including the outcomes of Cobb angle changes would also assist in determining the efficacy of the ScoliBrace^®^ and ScoliBalance^®^ interventions separately and in combination; however, this was not the aim of this current study. Also, with a large proportion of patients able to achieve the 180 s maximum cut-off, it may be worth applying the tests with >180 s, as applied by some authors [21].

## 5. Conclusions

This retrospective study shows a positive trend in improvements in trunk muscle endurance in AIS patients while using a combined treatment approach of ScoliBalance^®^ PSSE and ScoliBrace^®^. While the contribution of each treatment to trunk muscle endurance cannot be determined from this study, the results do indicate that trunk muscle endurance can be improved, and will likely not deteriorate, in AIS patients using ScoliBalance and ScoliBrace^®^. With the limited literature available, our study serves as part of the justification for including scoliosis-specific exercises with bracing treatment when indicated.

## Figures and Tables

**Table 1:** Description of initial dataset.

Total number of patients	33
Age	Mean 13.24 years Range 9–17 years Standard Deviation 1.64
Sex	Female 29 (87.88%) Male 4 (12.12%)
Primary Curve Location	Left Thoracolumbar 7 (21.21%) Right Thoracic 26 (78.79%)
Largest Cobb Angle	Mean 38.97° Standard Deviation 9.49

**Table 2:** Comparison between baseline and short-term and medium-term TE and AE in seconds, all cases.

Measure of Comparison	Baseline Median (*n*, Inter-Quartile Range)	Short-Term Median (*n*, Inter-Quartile Range)	Medium-Term Median (*n*, Inter-Quartile Range)	Median of the Difference (95% CI)	*p*-Value (Wilcoxon Signed- Rank Test)
TE ^1^	87 (33, 60–120)	150 (31, 81–180)	-	37 (18.49–55.01)	<0.001
TE ^1^	87 (33, 60–120)	-	180 (28, 133–180)	68.5 (46.83–92.50)	<0.001
TE ^1^	-	150 (31, 81–180)	180 (28, 133–180)	0 (0.00–39.34)	0.01
AE ^2^	95 (33, 76–180)	180 (32, 145–180)	-	33 (12.98–65.02)	<0.001
AE ^2^	95 (33, 76–180)	-	180 (29, 121–180)	47 (0.00–62.45)	0.002
AE ^2^	-	180 (32, 145–180)	180 (29, 121–180)	0 (0.00–15.05)	0.860

^1^ TE—Trunk extensor muscle endurance and ^2^ AE—Abdominal muscle endurance.

**Table 3:** Comparison between baseline and short-term and medium-term TE and AE in seconds in AIS who did not reach the maximum 180 s measurement.

Measure of Comparison	Baseline Median (*n*, Inter-Quartile Range)	Short-Term Median (*n*, Inter-Quartile Range)	Medium-Term Median (*n*, Inter-Quartile Range)	Median of the Difference (95% CI)	*p*-Value (Wilcoxon Signed-Rank Test)
TE ^1^	70 (27, 45–103)	94 (17, 74–133)	-	29 (16.05–48.90)	< 0.001
TE ^1^	70 (27, 45–103)	-	119 (12, 83–136.5)	47.5 (30.96–74.68)	0.002
TE ^1^	-	94 (17, 74–133)	119 (12, 83–136.5)	20.5 (−2.70–46.70)	0.025
AE ^2^	81.5 (24, 63–105.5)	131 (12, 67.5–152)	-	42.5 (14.49–75.72)	0.004
AE ^2^	81.5 (24, 63–105.5)	-	110 (11, 80–128)	45 (−36.21–58.14)	0.139
AE ^2^	-	131 (12, 67.5–152)	110 (11, 80–128)	24.5 (−40.80–70.50)	0.674

^1^ TE—Trunk extensor muscle endurance and ^2^ AE—Abdominal muscle endurance.

**Table 4:** Number and proportion of patients who reached 180 s at the three timepoints.

Measure	Timepoint	Total Number of Measurements	Number (Proportion) Who Did Not Reach 180 s	Number (Proportion) Who Reached 180 s
TE ^1^	Baseline	33	27 (81.82%)	6 (18.18%)
TE ^1^	Short-term	31	17 (54.84%)	14 (45.16%)
TE ^1^	Medium-term	28	12 (42.86%)	16 (57.14%)
AE ^2^	Baseline	33	24 (72.73%)	9 (27.27%)
AE ^2^	Short-term	32	12 (37.50%)	20 (62.50%)
AE ^2^	Medium-term	29	11 (37.93%)	18 (62.07%)

^1^ TE—Trunk Extensor Endurance and ^2^ AE—Abdominal Endurance.

**Figure A1:**
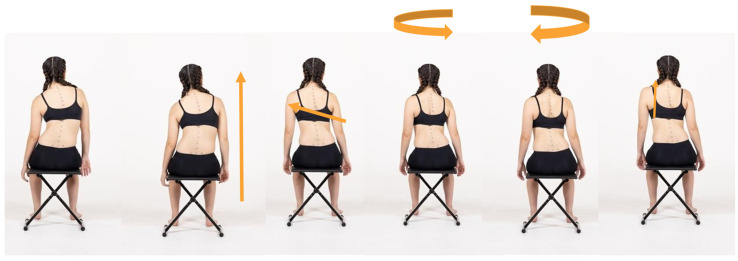
ScoliCorrection used in the ScoliBalance^®^ program using multiple steps to create the 3D auto-corrective movements. Photos used with permission from source.

**Figure A2:**
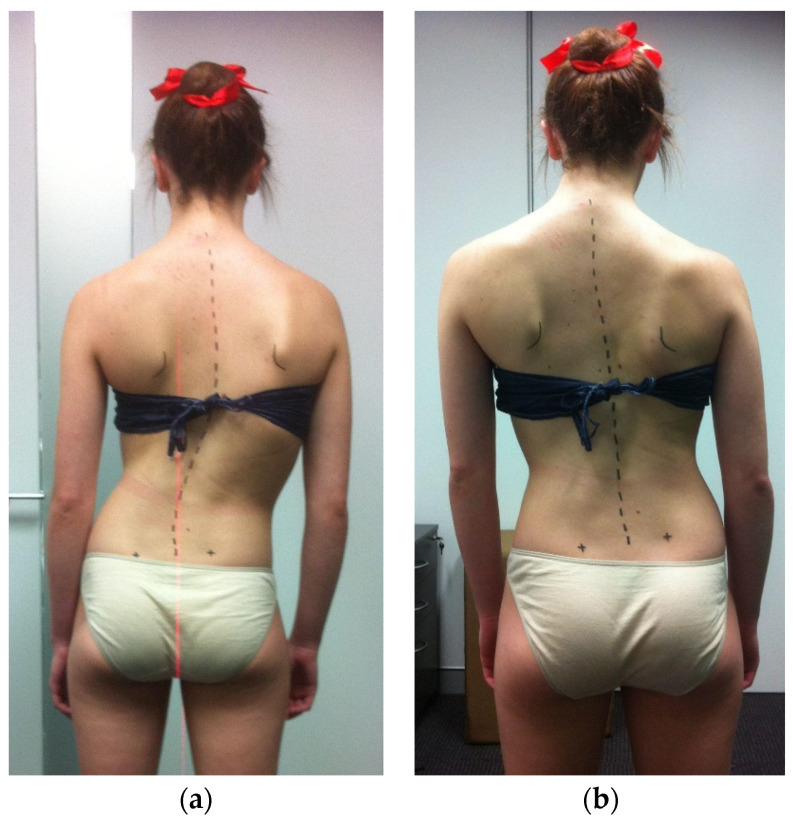
Photo depicting the demonstration of the patient with a right thoracic curve in a relaxed standing posture (**a**). The patient is then using ScoliBalance^®^ ScoliCorrections to perform an active 3D self-correction (**b**). Photos used with permission from source.

**Figure A3:**
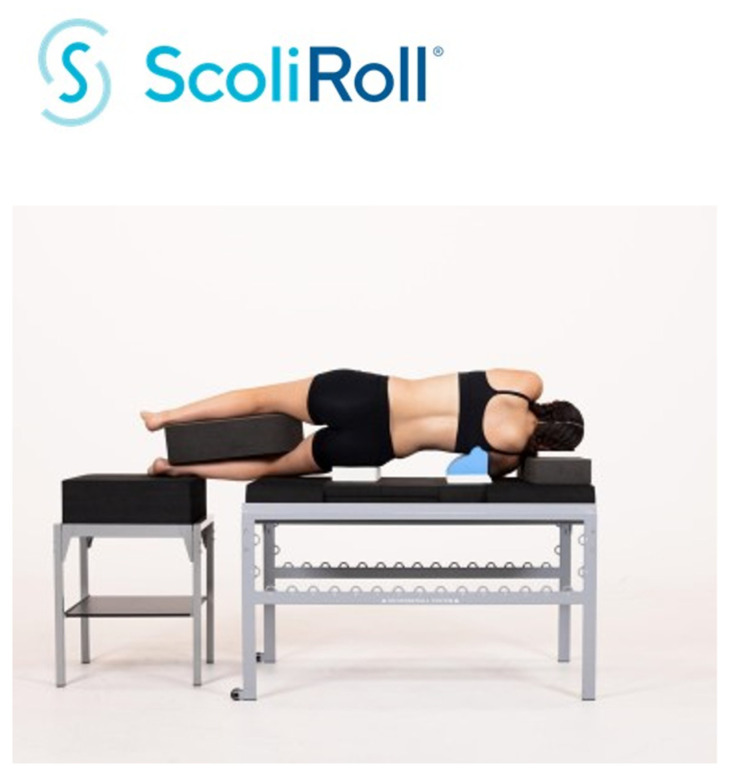
Demonstration of example positioning of the adolescent with idiopathic scoliosis undergoing the ScoliBalance^®^ program and including the prescription of the use of the ScoliRoll^®^ orthotic device. The patient was given this to use once per day at home for 25 min. Photos used with permission from source.

**Figure A4:**
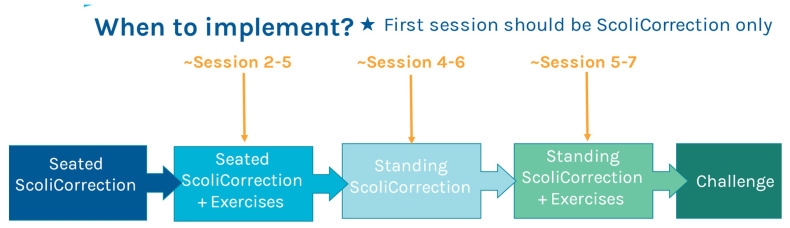
An example of the ScoliBalance^®^ ScoliCorrections and ScoliExercise progression over time from a seated to standing position, which is later challenged by exercises performed in the corrected position.

**Figure A5:**
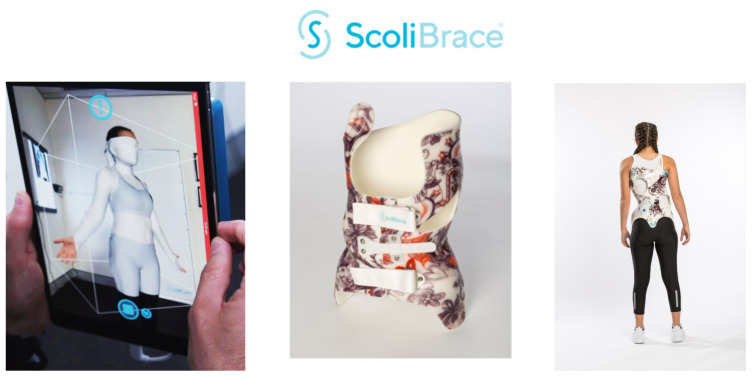
Photos used with permission from source.

## Data Availability

The data presented in this study are available on request from the corresponding author. The data are not publicly available due to privacy reasons.

## Appendix A. ScoliBalance^®^ Exercise Approach as a Physiotherapeutic Scoliosis-Specific Exercise (PSSE) Program for Adolescent Idiopathic Scoliosis (AIS)

### Appendix A.1. The Concept

Scoliosis creates 3D postural and structural changes in the spine and trunk, which can create postural imbalances. ScoliBalance^®^ is designed to help restore postural balance in the spine and trunk, maintain the integrity of balance and proprioception in the entire person, and reduce the Cobb angle of the curve if possible, or at least prevent its progression.

### Appendix A.2. Description of the ScoliBalance^®^ Program

To determine the ScoliBalance program for each AIS patient:

1. The curve classification was determined using a simple classification system, i.e., lumbar, thoracolumbar, thoracic, and double curve (primary thoracic, primary lumbar, or balanced).
2. The patient was prescribed a ScoliCorrection using multiple steps to create the corrective movements (Figure A1). This commenced in a sitting posture, whereby the patient was taught the steps of the ScoliCorrection with the aim of achieving 3D autocorrection. For single curves, the patient was prescribed a ScoliCorrection based on the curve classification and involved over-correction, mirror image^®^, and corrective breathing for posture correction [11,13,15,53]. The aim was to harness the biomechanical principles of spinal coupling through movement (Figure A2) where possible. Where overcorrection was not possible, e.g., due to large amounts of deformity, then the patient was instructed to focus more on achieving a ScoliCorrection in a neutral posture.
3. The patient was also given an orthotic (ScoliRoll^®^)device (Figure A3) to use once per day at home for 25 min, starting with 2 min on Day 1 and progressing to 25 min over 1–2 weeks.
4. The patient was then prescribed exercises to stabilize the corrected posture through ScoliExcercises and integration into activities of daily living, e.g., sit to stand. Progression over time is from a seated to standing position, which is later challenged by exercises in the corrected position (Figure A4).
5. Patient education was provided verbally to the patient at every session so that each day the patient was left able to independently complete the required exercises and integration into ADLs.
6. Each patient was prescribed 20–25 min of exercise daily. This incorporated an individualized approach, whereby each patient was prescribed a series of repetitions and sets of each exercise relevant to their curve type. The exercises were prescribed in a sitting position and progressed to standing by about week 5.
7. Each patient attended the clinic weekly and was asked to perform their home exercise program daily.
8. Compliance was monitored verbally at each weekly session. The program was progressed based on individual capacity and performance rather than a one-size-fits-all approach.

Patients were supervised through a series of postural corrections and exercises that progressed from passive correction to passive assisted corrections, active assisted corrections, and then on to active correction with the aim of moving on to 3D autocorrection. ‘Assistance’ was provided by the therapist’s hands where needed, until the patient was able to perform the exercises independently. Photographic feedback of the correction was provided to the patient.

Once the ScoliCorrection was taught and the patient was able to perform the correction as a 3D autocorrection, the patient was asked to complete a series of strengthening or stabilization exercises (ScoliExercises). See below for an example of the ScoliBalance^®^ ScoliExercise prescription where the patient was asked to hold their ScoliCorrection while performing the ScoliExercise (Table A1).

**Table A1:** Example of a ScoliBalance^®^ ScoliExercise prescription.

Session 1	Session 2	Session 3	Session 4	Session 5	Session 6	Sessions 7–9	Session 10+
Teach the appropriate ScoliCorrection step by step	Double arm raise (DA raise)	Seated bicep curl (BC)	Seated BC and shoulder press (SP)	Forward lean shoulder press	Standing Bicep curl	Standing BC Shoulder Press	Bent over row
Based on patient’s needs, goals, and clinical presentation	Seated ball toss	Seated T-row	Seated T to Y row	Lateral raise Band	Standing T-row	Standing T to Y row	Lunge
	Seated calf raise or leg lift	Seated marching	Seated Marching and Ball Toss	Two ball toss	Standing ball toss	Walking and ball toss	Tandem Walk Ball Toss
	Forward lean (FWD lean)	FWD lean DA or SA raise	Sit to stand	FWD/BWD lean + leg lift	Sit to Stand Squat	Squat	Squat Press
	Single arm raise (SA raise)	Backward lean (BWD lean)	Combined FWD/BWD lean	Standing ScoliCorrection	Standing calf raise	Calf Raise + Side Raise	Aeroplane
			Backward lean marching	BWD lean marching and ball toss	FWD/BWD lean + leg lift + arm lift	Step Up	Runner Step Up Curl

## Appendix B. ScoliBrace^®^

Every ScoliBrace^®^ (ScoliCare Pty Ltd. Sydney, Australia) is custom made for the individual by a dedicated design team and Computer-Aided Design and Manufacturer.

Unlike other traditional style scoliosis braces, which use a three-point pressure system, ScoliBrace^®^ uses an inverse corrective approach that harnesses spinal coupling to maximize correction where possible.

In most cases, ScoliBrace^®^ uses an over-corrective, mirror image^®^ approach to guide the body into a posture that is the opposite of the way the scoliosis has positioned it. By putting the body posture in this over-corrected position, it forces the spine to straighten up using the concept of spinal coupling, i.e., as the body moves into the opposite position, the spine moves with it towards that position, achieving the maximum straightening within the limits of the spine’s flexibility (Figure A5).
